# Primary Bone Tumors in Children and Adolescents Treated at a Referral Center in Northern Tanzania

**DOI:** 10.5435/JAAOSGlobal-D-17-00045

**Published:** 2019-03-05

**Authors:** Michelle Ghert, Winfrida Mwita, Faiton Ndesanjo Mandari

**Affiliations:** From the McMaster University, Hamilton, Ontario, Canada (Dr. Ghert), and the Kilimanjaro Christian Medical School, Moshi, Tanzania (Dr. Mwita and Dr. Mandari).

## Abstract

Bone tumors account for a small fraction of childhood cancers. Most published reports are from developed countries. The purpose of this study was to review the primary bone tumors in children and adolescents treated at a referral center in Northern Tanzania. We completed a 10-year hospital-based cross-sectional study in which all patients younger than 20 years diagnosed with a primary bone tumor at the Kilimanjaro Christian Medical Center Orthopaedic Department from January 2006 to December 2015 were identified and reviewed. Of the 80 identified patients, 15 (18.8%) were aged 5 to 8 years, and 65 (81%) were aged 9 to 19 years. Forty-seven males (59%) and 33 females (41%) were identified. The most common tumor locations were the femur, tibia, and humerus. Osteosarcoma was the most common malignant diagnosis (49 patients, 61%). No cases of Ewing sarcoma were reported. The most common tribal origins of the patients were Chagga and Maasai. Most primary bone tumors treated at a referral center in Northern Tanzania are malignant, with osteosarcoma representing the vast majority. No cases of Ewing sarcoma were identified in this tertiary referral hospital–based database.

Malignant bone tumors are rare in the human population. They exhibit a bimodal distribution in that a large proportion of bone sarcomas affect children and adolescents.^1^ It is estimated that bone sarcomas account for approximately 0.2% of all malignancies in the United States.^1^ Omololu et al^2^ reported that bone tumors represented 0.53% of all cancers seen in a hospital-based registry in Nigeria and 2.3% of the childhood cancers, with the most affected group being between ages 10 and 14 years. In a study from Uganda analyzing data from the 1960s, osteosarcoma was found to be the most common primary malignant bone tumor with a peak age of 10 to 19 years.^3^ However, further recent literature on bone tumors in Africa is scarce, given the low priority given to surgical oncology in Africa and the lack of cancer registries in most countries.^4^

Presentation with advanced stage osteosarcoma has been reported to be associated with a low socioeconomic status.^5^ In poor countries such as Tanzania, advanced presentation of disease and the lack resources for limb salvage generally result in the need for amputation, which is a notable burden on families and the ability of the patient to function in society.^6,7^ In less developed countries such as those in sub-Saharan Africa, standard treatment regimens may not be available or financially feasible, and logistic barriers to treatment lead to poor outcomes in pediatric cancer.^8^

The objective of this study was to review the pediatric and adolescent bone tumors treated at a referral center in Northern Tanzania to characterize the bone tumor distribution in this region.

## Methodology

### Study Design

We completed a 10-year hospital-based retrospective descriptive cross-sectional study in which all patients younger than 20 years diagnosed and/or treated for primary bone tumors at the Kilimanjaro Christian Medical Center (KCMC) Orthopaedic Unit from January 2006 to December 2015 were identified and reviewed. Patients seen in the outpatient clinic with benign and asymptomatic tumors were not admitted and therefore are not included in the database.

### Study Area

KCMC is located in Moshi, Tanzania. Moshi is a municipality in the Kilimanjaro region with a population of 184,292 (2012 census). Moshi covers approximately 59 square kilometers and is the smallest municipality in Tanzania by area. However, KCMC is one of the four recognized referral hospitals in Tanzania. The hospital receives patients from the Kilimanjaro region and its neighboring regions in the Northern zone and other parts of the United Republic of Tanzania, in addition to patients from neighboring countries including Kenya. KCMC is a large complex with 500 to 800 inpatients in 630 official beds, 40 neonatal incubators, 1852 students, 1300 staff, and 1000 patient visitors and companions daily. The KCMC Orthopaedic Department has 53 staff including attending surgeons and residents.

### Study Population

All patients admitted and treated for a primary bone tumor at the KCMC Orthopaedic Department between January 1, 2006, and December 31, 2015, were identified through prospectively maintained admission records. Patients older than 19 years were excluded.

### Data Extraction

A data collection sheet was developed and transcribed onto an electronic data file. Data collected included primary bone tumor diagnosis, sociodemographic characteristics (ie, age, sex, area of residence, tribe, and occupation), and site of bone tumor (ie, bone involved). Because of the lack of resources required for a secondary pathology review, the tissue slides were not pulled and reviewed a second time. The data were collected from the pathology reports at the time of diagnosis.

### Data Processing and Analysis

Data were processed and analyzed using Statistical Package for the Social Sciences, version 2.0. Descriptive statistics were used to summarize the data. For continuous variables, data were summarized using mean with corresponding measures of dispersion. For categoric variables, frequencies and percentages were used.

### Ethical Considerations

Ethical clearance was received from the Kilimanjaro Christian Medical Centre Orthopaedic Department through the Community Health Department. Confidentiality was maintained throughout the study whereby no unauthorized person was allowed to access any data collected. Hospital registration numbers were used instead of patients' names during the study.

## Results

### Demographic Characteristics

A total of 80 patients met the inclusion criteria. The demographic characteristics of the patients are shown in Table 1. Of the 80 patients treated for a primary bone tumor at KCMC from year 2006 to 2015, 15 patients (18.8%) were aged 5 to 8 years, whereas 65 patients (81.3%) were aged 9 to 19 years. No patients younger than 5 years were identified. The mean age at the time of diagnosis was 12 years. Forty-seven males (58.8%) and 33 females (41.3%) were identified, resulting in a male:female ratio of 1.4:1. Sixty-four patients (80%) were students at the time of diagnosis, whereas the remaining 16 patients (20%) were children who had not yet started school.

**Table 1:**
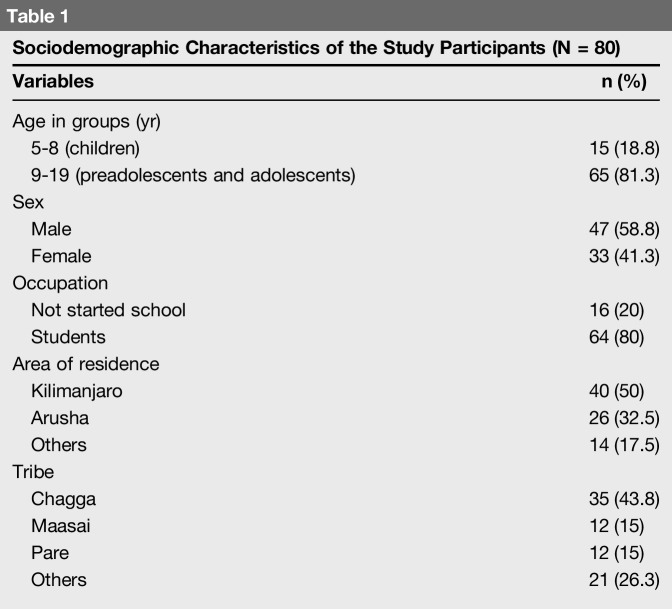
Sociodemographic Characteristics of the Study Participants (N = 80)

Most patients resided in the Kilimanjaro region (40 patients, 50%) and the Arusha region (26 patients, 32.5%). Fourteen patients (17.5%) resided in other regions. The most common tribal origin of the patients was Chagga (35 patients, 43.8%), whereas 12 patients (15% each) were Maasai and Pare. Other tribes constituted 26.3% (21 patients).

### Anatomic Location

Regarding anatomic distribution, the long bones of the lower extremity were most commonly affected, with 32 tumors (40%) in the femur and 7 (8.8%) in the tibia. Other locations included the humerus (6 tumors, 7.5%), phalanges (4 tumors, 5%), scapula (3 tumors, 4%), and clavicle (3 tumors, 4%). The vertebral column, calcaneus, and talus were affected in one patient each. Data regarding location within the bone are not available.

### Histologic Diagnosis

The histologic diagnoses according to the World Health Organization 2002 classifications are shown in Table 2. Most primary bone tumors were malignant (71%). The most common diagnosis was osteosarcoma (49 patients, 61.2%) and chondrosarcoma (8 patients, 10%). Benign diagnoses included osteochondroma (9 patients, 11.2%), giant cell tumor, and cavernous hemangioma (3 patients, 3.8% each). Other less common diagnoses included chondromyxoid fibroma, enchondroma, chondroblastoma, fibrous histiocytoma, and solitary bone cyst. No cases of Ewing sarcoma of bone were identified from the database during the study period. We found a relatively high percentage of chondrosarcoma in this young population (10%). This phenomenon may be related to the need for a specialized pathologist, which is an important challenge for resource-strained environments such as Tanzania. It is possible that the specimens were chondroid subtypes of osteosarcoma, but subtyped as chondrosarcoma instead.

**Table 2:**
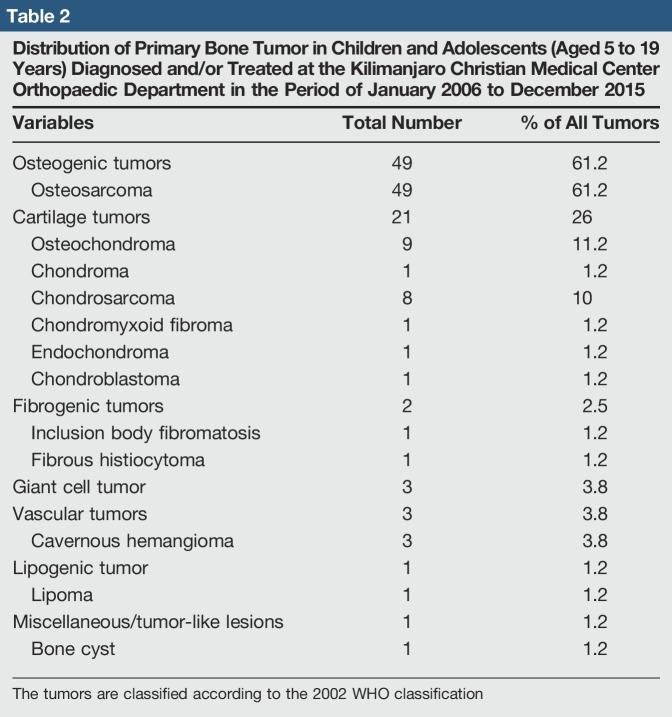
Distribution of Primary Bone Tumor in Children and Adolescents (Aged 5 to 19 Years) Diagnosed and/or Treated at the Kilimanjaro Christian Medical Center Orthopaedic Department in the Period of January 2006 to December 2015

## Discussion

### Summary of Findings

Osteosarcoma was the most common primary bone tumor diagnosed in children and adolescents at KCMC during the study period. The long bones of the extremities were the most affected sites, with the femur being the most common, whereas the vertebral column, talus, and calcaneus were the least affected sites. In terms of age and sex, males were more affected than females, and preadolescents and adolescents (9 to 19 years) were more affected than children (5 to 8 years). The mean age at diagnosis was 12 years. There was a notable absence of patients diagnosed with Ewing sarcoma.

### Findings in Relation to Previous Studies

The more common occurrence of bone tumors in preadolescents and adolescents as opposed to children is consistent with previous reports. The frequency of occurrence corresponds with the period of peak skeletal growth, which is reported to occur at age 12 years for females and 14 years for males.^9^ In the current study, males were more affected than females with primary bone tumors at a ratio of 1.4:1. This phenomenon is identical to the ratio reported in Nigeria by Omololu et al^2^ where males accounted for 58.8% of the cases seen and females accounted for 41.2% of the cases (ratio of 1.4:1).

As a group, primary bone tumors have a propensity to originate in the long bones of the extremities.^9^ In this study, 56% of the tumors were located in the long bones. This phenomenon is similar to what has been reported in Nigeria where 47.3% of bone tumors were located in the long bones.^2^ We identified the femur as the most common tumor location in our center (40%), which corresponds to data from a single center in Europe where the femur was the most common location and accounted for 26.7% of affected bones^10^ and with data from the United States (25%) and China (42.3%) where the femur was also the most common location.^11^

Osteosarcoma was the most common bone tumor identified in this study, and this correlates with findings from Nigeria^2^ and Ethiopia.^12^ However, unlike studies from Europe,^10^ China,^11^ Nigeria,^2^ and Ethiopia,^12^ Ewing sarcoma was not seen in this study. Data from Nigeria also report a lower incidence of Ewing sarcoma (0.9%).^2^ Ewing sarcoma is associated with a specific cytogenetic translocation t(11;22)(q24;q12)^13^ and is generally uncommon in black African children and almost exclusively limited to Caucasians.^14^ Studies from North America and other countries confirm the relatively low incidence of Ewing sarcoma in the black population.^15,16^ In this study, all the cases were black Africans.

### Limitations

This is a retrospective study, and as data were collected, it was noted that some of the hospital registration books were missing some pages and some data could not be assessed (recall bias). In addition, the cases that were recorded in the hospital registration books were for those who were admitted to the KCMC Orthopaedic Department, so those patients seen the outpatient clinic without being admitted were included (selection bias). However, those not admitted were unlikely to present with clinically significant bone tumor findings and therefore did not require inpatient intervention. Many patients in Tanzania present with advanced stage disease as they seek a local healer first. Although we are not able to report specific staging information, most patients in this context present with metastases and succumb to their disease. Although we did not identify any patients with Ewing sarcoma, it is possible that they were missed if pathology was not available. However, this is true of all other tumor subtypes. Finally, this study is not a population-based study because the data were obtained from a single hospital, albeit a referral center for a large area of East Africa.

However, this study presents a diverse population from a large region in East Africa. It is the first such report from the United Republic of Tanzania and represents cross-sectional data of a referral center serving a unique population in East Africa. Most primary bone tumors treated at a referral center in Northern Tanzania are malignant, with osteosarcoma representing the vast majority. No cases of Ewing sarcoma were identified in this tertiary referral hospital–based database, which may reflect the ethnic background of the surrounding population.

## References

[1] Franchi A: Epidemiology and classification of bone tumors. Clin Cases Miner Bone Metab 2012;9:92-995. 23087718 PMC3476517

[2] Omololu AB Okolo CA Ogunlade SO: Primary malignant bone tumours in Ibadan, Nigeria: An update. Afr J Med Med Sci 2009;38:77-81. 19722432

[3] Dodge OG: Tumours of bone and jaw. Recent Results Cancer Res 1973;41:222-233. 4373802 10.1007/978-3-642-80725-1_14

[4] Hadley LG Rouma BS Saad-Eldin Y: Challenge of pediatric oncology in Africa. Semin Pediatr Surg 2012;21:136-141. 22475119 10.1053/j.sempedsurg.2012.01.006

[5] Duchman KR Gao Y Miller BJ: Prognostic factors for survival in patients with high-grade osteosarcoma using the Surveillance, Epidemiology, and End Results (SEER) Program database. Cancer Epidemiol 2015;39:593-5999. 26002013 10.1016/j.canep.2015.05.001

[6] Wamisho BL Admasie D Negash BE Tinsay MW: Osteosarcoma of limb bones: A clinical, radiological and histopathological diagnostic agreement at black lion teaching hospital, Ethiopia. Malawi Med J 2009;21:62-65. 20345006 10.4314/mmj.v21i2.44552 PMC3345732

[7] Mosaku KS Akinyoola AL Fatoye FO Adegbehingbe OO: Psychological reactions to amputation in a sample of Nigerian amputees. Gen Hosp Psychiatry 2009;31:20-24. 19134505 10.1016/j.genhosppsych.2008.08.004

[8] Slone JS Chunda-Liyoka C Perez M: Pediatric malignancies, treatment outcomes and abandonment of pediatric cancer treatment in Zambia. PLoS One 2014;9:e89102. 24586527 10.1371/journal.pone.0089102 PMC3931678

[9] Ottaviani G Jaffe N: The epidemiology of osteosarcoma. Cancer Treat Res 2009;152:3-13. 20213383 10.1007/978-1-4419-0284-9_1

[10] Bergovec M Kubat O Smerdelj M Seiwerth S Bonevski A Orlic D: Epidemiology of musculoskeletal tumors in a national referral orthopedic department: A study of 3482 cases. Cancer Epidemiol 2015;39:298-302. 25703268 10.1016/j.canep.2015.01.015

[11] Niu X Xu H Inwards CY: Primary bone tumors: Epidemiologic comparison of 9200 patients treated at Beijing Ji Shui Tan hospital, Beijing, China, with 10 165 patients at Mayo Clinic, Rochester, Minnesota. Arch Pathol Lab Med 2015;139:1149-1155. 25978765 10.5858/arpa.2014-0432-OA

[12] Negash BE Admasie D Wamisho BL Tinsay MW: Pattern of bone tumours seen at Addis Ababa University, Ethiopia. East Cent Afr J Surg 2009;14:25-32.

[13] Bernstein M Kovar H Paulussen M: Ewing's sarcoma family of tumors: Current management. Oncologist 2006;11:503-519. 16720851 10.1634/theoncologist.11-5-503

[14] Akang EEU: Tumors of childhood in Ibadan, Nigeria (1973-1990). Pediatr Pathol Lab Med 1996;16:791-800. 9025877

[15] Jawad MU Cheung MC Min ES Schneiderbauer MM Koniaris LG Scully SP: Ewing sarcoma demonstrates racial disparities in incidence-related and sex-related differences in outcome. Cancer 2009;115:3526-3536. 19548262 10.1002/cncr.24388

[16] Parkin DM Stiller CA Nectoux J: International variations in the incidence of childhood bone tumours. Int J Cancer 1993;53:371-376. 8428791 10.1002/ijc.2910530305

